# Functional molecules in mesothelial‐to‐mesenchymal transition revealed by transcriptome analyses

**DOI:** 10.1002/path.5101

**Published:** 2018-07-04

**Authors:** Sara Namvar, Adrian S Woolf, Leo AH Zeef, Thomas Wilm, Bettina Wilm, Sarah E Herrick

**Affiliations:** ^1^ Division of Cell Matrix Biology and Regenerative Medicine, School of Biological Sciences, Faculty of Biology, Medicine and Health University of Manchester Manchester UK; ^2^ Manchester Academic Health Science Centre Manchester UK; ^3^ Royal Manchester Children's Hospital Manchester University NHS Foundation Trust Manchester UK; ^4^ The Bioinformatics Core Facility The University of Manchester Manchester UK; ^5^ Institute of Translational Medicine University of Liverpool Liverpool UK

**Keywords:** bone morphogenetic protein, insulin‐like growth factor, peritoneum, mesothelium

## Abstract

Peritoneal fibrosis is a common complication of abdominal and pelvic surgery, and can also be triggered by peritoneal dialysis, resulting in treatment failure. In these settings, fibrosis is driven by activated myofibroblasts that are considered to be partly derived by mesothelial‐to‐mesenchymal transition (MMT). We hypothesized that, if the molecular signature of MMT could be better defined, these insights could be exploited to block this pathological cellular transition. Rat peritoneal mesothelial cells were purified by the use of an antibody against HBME1, a protein present on mesothelial cell microvilli, and streptavidin nanobead technology. After exposure of sorted cells to a well‐known mediator of MMT, transforming growth factor (TGF)‐β1, RNA sequencing was undertaken to define the transcriptomes of mesothelial cells before and during early‐phase MMT. MMT was associated with dysregulation of transcripts encoding molecules involved in insulin‐like growth factor (IGF) and bone morphogenetic protein (BMP) signalling. The application of either recombinant BMP4 or IGF‐binding protein 4 (IGFBP4) ameliorated TGF‐β1‐induced MMT in culture, as judged from the retention of epithelial morphological and molecular phenotypes, and reduced migration. Furthermore, peritoneal tissue from peritoneal dialysis patients showed less prominent immunostaining than control tissue for IGFBP4 and BMP4 on the peritoneal surface. In a mouse model of TGF‐β1‐induced peritoneal thickening, BMP4 immunostaining on the peritoneal surface was attenuated as compared with healthy controls. Finally, genetic lineage tracing of mesothelial cells was used in mice with peritoneal injury. In this model, administration of BMP4 ameliorated the injury‐induced shape change and migration of mesothelial cells. Our findings demonstrate a distinctive MMT signature, and highlight the therapeutic potential for BMP4, and possibly IGFBP4, to reduce MMT. © 2018 The Authors. *The Journal of Pathology* published by John Wiley & Sons Ltd on behalf of Pathological Society of Great Britain and Ireland.

## Introduction

Epithelia form sheets and tubules conferring physical integrity and performing physiological functions. Epithelial‐to‐mesenchymal transition (EMT) occurs in normal development during gastrulation and neural crest migration. EMT is characterized by disrupted cell–cell adhesion and apical–basolateral polarity, cytoskeletal reorganization, detachment from basement membranes, and the generation of motile mesenchymal cells. The reverse process, mesenchymal‐to‐epithelial transition, occurs during somitogenesis and nephrogenesis 1, 2. Mesothelial cells (MCs) are epithelial‐like cells lining the coelomic cavities and the organs that they contain. MCs have junctional complexes and apical–basolateral polarity, and adhere to a basement membrane. MCs secrete glycosaminoglycans and surfactant, permitting frictionless gliding of organs, and act as a barrier expressing inflammation‐modulating cytokines. MCs *in vivo* not only express cytokeratins, which is characteristic of epithelia, but also vimentin, which is more typical of mesenchyme 3. In normal development, some MCs undergo mesothelial‐to‐mesenchymal transition (MMT) to form vascular smooth muscle 4, 5. In development and cancer, the transcription factors Snail, Twist and Slug drive EMT 6, 7. MCs are not typical epithelial cells, so the biological characteristics of MMT and EMT may not be identical.

Fibrosis is an aberrant response to injury, and therapies to slow or reverse fibrosis are urgently needed. α‐Smooth muscle actin (αSMA)‐expressing myofibroblasts drive fibrosis, and it has been proposed that EMT generates some of these cells 1, 8, 9. In response to injury, MCs can undergo MMT 10, 11, 12, 13. For example, after injection of labelled MCs into the peritoneal cavity, they appear in the regenerating mesothelial layer and in the submesothelial layer 14. Peritoneal fibrosis can be triggered by peritoneal dialysis, causing treatment failure, and by surgery, causing adhesions. Targeting of MMT may therefore prevent scarring. Mesothelial damage by peritoneal dialysis or surgery initiates the production of profibrotic mediators, notably transforming growth factor (TGF)‐β1 3. We hypothesized that, if the molecular signature of TGF‐β1‐induced MMT could be defined, these insights could be exploited to ameliorate MMT.

## Materials and methods

### Animals

Animal experiments were undertaken according to ARRIVE guidelines, and were approved by the Review Boards of the Universities of Manchester and Liverpool, and by the Home Office.

### Isolation, purification and culture of rat MCs

Omental tissue was dissected from 9–12‐week‐old female Wistar rats weighing 220–250 g (Charles River, Harlow, UK). Tissue was dissociated in 0.25% trypsin–EDTA (Sigma‐Aldrich, Gillingham, UK) for 20 min at 37 °C. Cells were incubated with HBME1 antibody (M3505, 1:50; Dako, Cambridge, UK) for 30 min in 3% bovine serum albumin in phosphate‐buffered saline (PBS). Cells were washed and incubated for 30 min with biotinylated secondary antibody (BA2020, 1:100; Vector Laboratories, Peterborough, UK). Following further washes, 1.5 × 10^6^ cells were incubated with streptavidin‐coated magnetic nanobeads (Biolegend, London, UK) for 15 min, and then placed in a MojoSort magnet (Biolegend) for 5 min. Uncaptured cells were decanted, and the remainder were resuspended in culture media comprising high‐glucose Dulbecco's modified Eagle's medium supplemented with 15% fetal bovine serum (FBS), 4 mm l‐glutamine (Sigma‐Aldrich), 1% v/v penicillin/streptomycin, and 0.4 μg/ml hydrocortisone (Sigma‐Aldrich). In other experiments, cells from trypsinized omentum were subjected to fluorescence‐activated cell sorting (FACS), as described previously 15. Cells were processed as for magnetic bead sorting, but HBME1 antibody was detected with IgM Alexa488 (A‐21042, 1:1000; Thermo Fisher Scientific, Runcorn, UK). Cells were seeded at 5 × 10^4^/cm^2^ in multiwell plates. After comparison of the two methods, subsequent experiments were performed with Mojo purification. After 48–72 h of culture following enrichment, cells were washed with Hanks' balanced salt solution, and the medium was changed every other day for up to 10–11 days. Cells were then placed in low‐serum (5% FBS) medium. Cells were exposed to 1 ng/ml TGF‐β1 for 48 h (R&D Systems, Abington, UK) and/or 50 ng/ml bone morphogenetic protein (BMP) 4 (Biolegend) and/or 50 ng/ml insulin‐like growth factor (IGF)‐binding protein (IGFBP) 4 (Biolegend).

### RNA sequencing (RNA‐seq) and quantitative polymerase chain reaction (qPCR)

These are detailed in supplementary material, Supplementary materials and methods. For RNA‐seq, differentially expressed transcripts were defined as those for which the log_2_(fold change) was ≥0.36 or ≤ −0.36 versus controls, and for which there was a statistical significance of *p* < 0.05, corrected for multiple comparisons. (Fold change = test value/control value.) RNA‐seq data are available at ArrayExpress E‐MTAB‐5998.

### Immunostaining, cell migration assay, and enzyme‐linked immunosorbent assay (ELISA)

Details are given in supplementary material, Supplementary materials and methods.

### TGF‐β1‐induced peritoneal fibrosis and peritoneal MC lineage tracing in mice

Two models of peritoneal injury were studied. In the first, tissues from wild‐type C57BL/6 J mice that had received intraperitoneal adenovirus expressing TGF‐β1 were analysed; we previously reported that this led to submesothelial fibrosis *in vivo* 16. In a second model, we combined surgical abrasion of the peritoneum 17 with an MC lineage tracing strategy, similar to that described by Lua *et al* 18, but using *LacZ* rather than *GFP*. Detailed protocols are given in supplementary material, Supplementary materials and methods.

### Human tissue analyses

After informed consent and ethical approval (REC 06/Q1407/94 and 14/NE/0059), peritoneum samples were collected from patients undergoing hernia repair (*n* = 4) and from end‐stage kidney disease patients who had undergone peritoneal dialysis (*n* = 4). Tissues were fixed in 4% paraformaldehyde for 24 h, and processed into paraffin blocks, from which 7‐μm sections were taken and permeabilized with 0.2% Triton in PBS, and then incubated with primary antibodies against HBME1 (Dako; M3505, 1:50), IGFBP4 (Abcam Cambridge, UK; ab83846, 1:700) or BMP4 (Abcam; ab39973, 1:100) overnight at 4 °C. Sections were incubated with the Rabbit IgG Specific horseradish peroxidase/3,3′‐diaminobenzidine (ABC) detection kit (Abcam). Images were obtained with a light microscope (Olympus, Southend‐on‐Sea, UK) and Image‐Pro Plus software (Media Cybernetics, Cambridge, UK).

### Statistics

Analysis for RNA‐seq data is outlined above. All other datasets were normally distributed, and so are presented as mean ±standard error of the mean (SEM). Student's *t*‐tests or anova with Tukey *post hoc* tests were used to compare groups. Analyses were performed with GraphPad Prism 6 (GraphPad Software, La Jolla, CA, USA).

## Results

### MC enrichment

**Figure 1:**
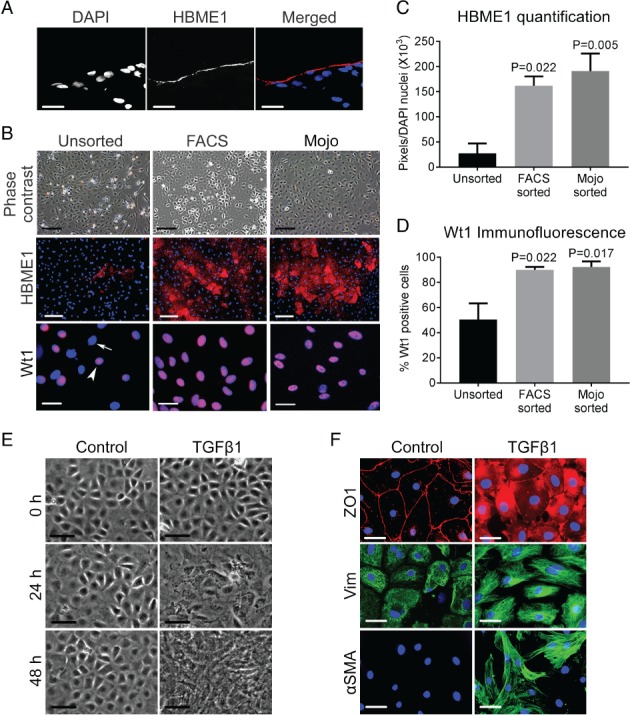
MC enrichment with HBME1 as a surface marker. (A) Fluorescence microscopy of rat omentum, showing all nuclei stained with 4′,6‐diamidino‐2‐phenylindole (DAPI), the MC apical surface immunostained for HBME1, and a merged overlay image. Scale bars: 50 μm. (B) Phase contrast and immunofluorescence images of primary cultures of unsorted, HBME1 FACS‐sorted and HBME1 magnetic bead (Mojo)‐sorted cells; note the prominent cobblestone phenotype of the sorted cells. Scale bars: 200 μm. Relative to unsorted cells, both FACS‐sorted and Mojo‐sorted cells are markedly enriched for HBME1. Note the presence of both Wt1‐positive (arrowhead) and Wt1‐negative (arrow) nuclei in the unsorted population stained with DAPI. Scale bars: 50 μm. (C) HBME1 enrichment was confirmed by measuring the pixels of positive immunostaining normalized to DAPI nuclei in FACS‐sorted and Mojo‐sorted cells (*n* = 6; mean ±SEM). (D) The percentage of Wt1‐positive nuclei was increased following either FACS or Mojo sorting (*n* = 3; mean ±SEM). (E) Cells were cultured for 48 h with or without 1 ng/ml TGF‐β1. Note the disruption of the cobblestone phenotype under TGF‐β1 treatment, with cells becoming elongated. Scale bars: 50 μm. (F) Fluorescence microscopy of cells at 48 h, demonstrating that exposure to TGF‐β1 was associated with disruption of the reticular cell–cell junctional ZO1 pattern, with more prominent cytoplasmic immunostaining for vimentin (Vim) and αSMA. Nuclei were stained with DAPI. Scale bars: 50 μm.

In rat omentum sections, HBME1 was immunodetected in MCs (Figure 1A), but adipose and connective tissue were negative. Primary cultures of omental cells that had been selected after binding to the HBME1 antibody either by FACS or magnetic bead sorting (Mojo) showed an enhanced cobblestone appearance versus unsorted cells (Figure 1B). FACS and magnetic bead sorting each resulted in significant enrichment of cells expressing HBME1 (Figure 1B,C) or Wt1 (Figure 1B,D), an MC transcription factor 4, 5. Unsorted cells showed a mean HBME1 immunostaining level of ≈27 000 pixels/nucleus, which rose after HBME1 FACS to 162 000 (*p* = 0.022) or after magnetic bead sorting to 192 000 (*p* = 0.005). Wt1 was immunodetected in approximately half of all nuclei of unsorted cells, rising to 90% after HBME1 FACS sorting (*p* = 0.022) and to 92% after HBME1 magnetic bead sorting (*p* = 0.017).

### Induction of MMT in cell culture

Mojo‐sorted MCs were exposed to TGF‐β1, a driver of both MMT and peritoneal fibrosis 19, 20. After 48 h, untreated MCs maintained their cobblestone morphology (Figure 1E), whereas parallel cultures exposed to 1 ng/ml TGF‐β1 progressively lost their epithelioid appearance and acquired an irregular elongated morphology (Figure 1E). Primary cultures of sorted cells showed positive immunostaining for ZO1, a tight junction protein, and vimentin, an intermediate filament protein (Figure 1F), as expressed by MCs *in vivo* 21, 22. Immunostaining for E‐cadherin was barely detectable, whereas MCF7 breast epithelial cells showed prominent cell–cell junction immunostaining (supplementary material, Figure S1). On immunohistochemistry, the mesothelial layer of rat omentum *in vivo* was negative for E‐cadherin, whereas nearby pancreatic ductal cells were positive (supplementary material, Figure S1). In TGF‐β1‐exposured Mojo‐sorted MCs: ZO1 became less prominent at cell–cell junctions, instead appearing in a cytoplasmic pattern; vimentin became more prominent; and αSMA, a smooth muscle contractile protein, became upregulated versus untreated cells (Figure 1F). Therefore, this protocol induced certain phenotypic changes considered to be typical of MMT 23, 24.

### Gene expression in purified MCs

**Figure 2:**
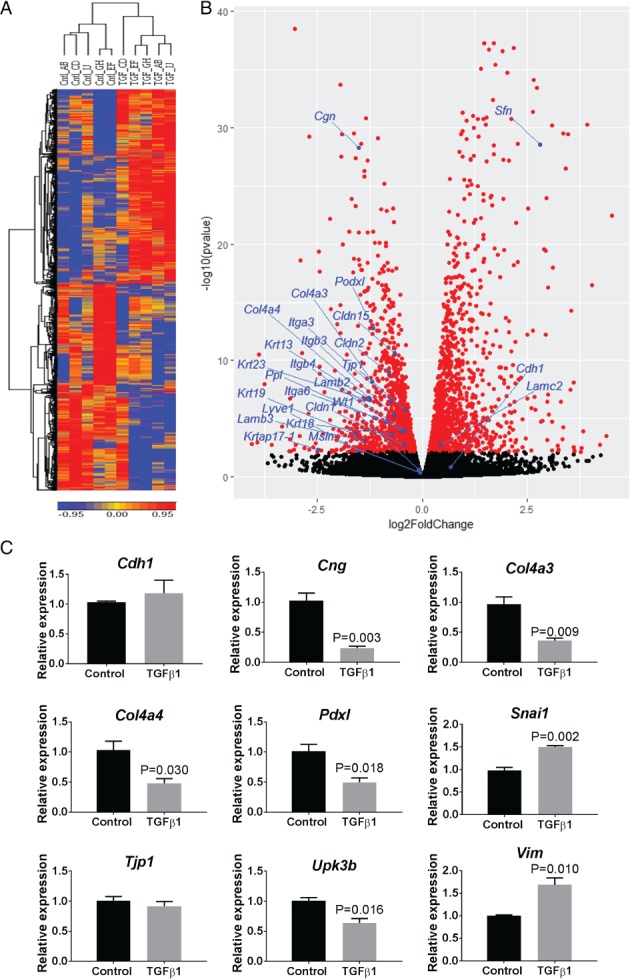
Transcriptome analyses of HBME1‐sorted rat MCs. (A) Unsupervised hierarchical clustering by transcript expression. Rows are expression levels denoted as the *z*‐score, displayed in a high–low (red–blue) colour scale; the numeric scale indicates *z*‐transformation. Note that levels of numerous transcripts are increased or decreased after 48 h of exposure to 1 ng/ml TGF‐β1. Cntl indicates the five vehicle‐only exposed samples. TGF indicates the five parallel cultures exposed to TGF‐β1. (B) Selected RNA‐seq data displayed as a volcano plot, with the image annotated for ‘epithelial signature’ transcripts. (C) qPCR for *Cdh1*, *Col4a3*, *Col4a4*, *Cng*, *Pdxl*, *Snai1*, *Tjp1*, *Upk3b*, and *Vim* (*n* = 3; mean ±SEM).

To define the transcriptome of purified MCs, we undertook RNA‐seq. In MCs exposed to 1 ng/ml TGF‐β1 for 48 h, the levels of 834 transcripts increased, and the levels of 487 transcripts were decreased, versus cells cultured without exogenous TGF‐β1. Unsupervised hierarchical clustering clearly distinguished between the two groups (Figure 2A). The volcano plot in Figure 2B is annotated for changed ‘epithelial signature’ transcripts; changed transcripts in this class are listed in supplementary material, Table S1. Among downregulated transcripts were: *Cng*, encoding the tight junction protein cingulin; *Cldn2* and *Cldn15*, encoding tight junction claudins; *Col4a3* and *Col4a4*, encoding epithelial basement membrane collagens; *Itga3*, *Itga6*, *Itgb3*, and *Itgb4*, encoding integrins; *Krt13*, *Krt18*, *Krt19*, and *Krt23*, encoding keratin intermediate filaments; *Lamb2* and *Lamb3*, encoding laminin B2 and laminin 3; *Podxl*, encoding sialomucin podocalyxin‐like protein 1; *Ppl*, encoding the desmosomal protein periplakin; and *Upk3b*, encoding uroplakin 3B, a plasma membrane protein that is characteristic of mesothelia *in vivo* 25. Control cells expressed high levels of *Wt1*, and *Msln*, encoding the glycosylphosphatidylinositol‐anchored cell surface protein mesothelin 26, but only low levels of *Cdh1*, encoding the cell–cell adhesion protein, E‐cadherin. Moreover, levels of these three transcripts did not significantly change upon TGF‐β1 exposure. Notably, *Sfn* levels rose after exposure to TGF‐β1; this transcript encodes stratifin, which has been linked to epithelial differentiation 27. qPCR was undertaken (Figure 2C) for a subset of transcripts (*Cdh1*, *Cng*, *Col4a3*, *Col4a4*, *Pdxl*, *Snai1*, *Tjp1*, *Upk3b*, and *Vim*), with generally similar findings to those of RNA‐seq, although the decrease in the level of *Tjp1* was not significant. Notably, *Cdh1* was detectable, but at a very low level by RNA‐seq, and its level was unchanged by TGF‐β1 (supplementary material, Table S1). E‐cadherin, the encoded protein, was not detected *in vivo*, and was barely detected in cultured MCs (supplementary material, Figure S1).

A selection of ‘mesenchymal/extracellular matrix signature’ transcripts is shown in supplementary material, Table S2. TGF‐β1 exposure led to increases in the levels of *Acta2*, encoding αSMA, and *Vim*, encoding vimentin. These increases, however, were not as great as those for the following transcripts: *Ncam1* and *Vcam1*, encoding neural and vascular cell adhesion molecules, respectively; and *Tnc* and *Tnn*, encoding, respectively, tenascin C and tenascin N, both of which are extracellular matrix glycoproteins. Transcripts previously implicated in classic EMT are listed in supplementary material, Table S3. Among them, the following transcripts were upregulated: *Tgfb1*, *Tgfb2*, and *Tgfb3*; and *Snai1* and *Snai2*, encoding snail zinc finger proteins 1 and 2. We found that the levels of *Twist1* and *Twist2*, encoding transcription factors considered to be key effectors of classic EMT 1, 28, were not significantly altered by TGF‐β1 (supplementary material, Table S3).

### MMT is associated with altered transcripts of BMP and IGF pathway molecules

**Figure 3:**
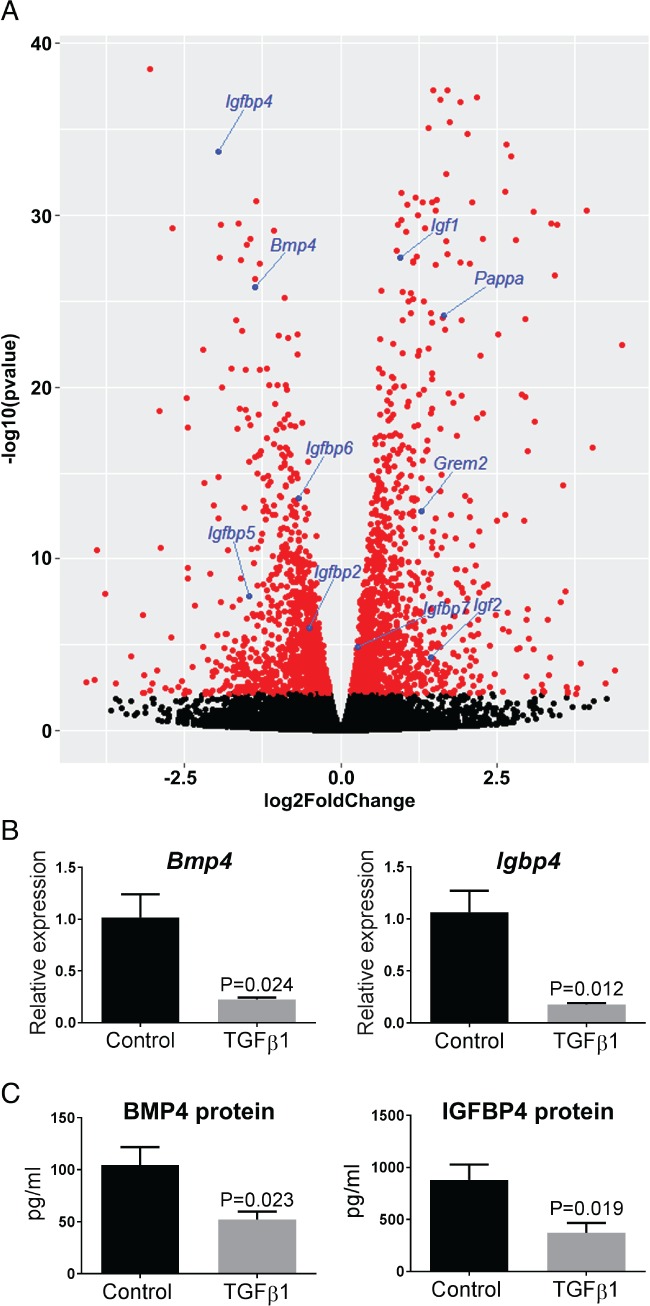
MMT is associated with dysregulation of the BMP4 and IGF pathways. (A) Volcano plot annotated with transcripts encoding molecules implicated in BMP and IGF signalling. (B) Confirmation of decreased levels of *Bmp4* and *Igfbp4* as assessed by qPCR (*n* = 3; mean ±SEM). (C) As determined by ELISA, the levels of BMP4 and IGFBP4 were decreased in medium conditioned by cells exposed to 1 ng/ml TGF‐β1 (*n* = 5; mean ±SEM).

RNA‐seq (Figure 3A; supplementary material, Table S4) revealed that control MCs expressed high levels of *Bmp4* but low levels of *Bmp7*. *Bmp4* was markedly downregulated upon exposure to TGF‐β1, whereas the levels of *Grem2*, which encodes the BMP antagonist gremlin 2 29, increased markedly, as did those of *Bmp1*, encoding an atypical BMP family member that is a secreted metalloprotease implicated in cartilage formation 30. TGF‐β1 exposure led to increases in the levels of *Igf1* and *Igf2*, encoding, respectively, IGF1 and IGF2. The levels of *Igfbp2*, *Igfbp4*, *Igfbp5* and *Igfbp6* decreased. These encode IGFBPs that alter the interaction of IGFs with their cell surface receptors, usually decreasing IGF signalling 31. We also detected markedly increased levels of *Pappa*, which encodes pregnancy‐associated plasma protein A, a secreted metalloproteinase that cleaves IGFBPs, rendering them inactive 32. As assessed by qPCR, the levels of *Bmp4* and *Igfb4* transcripts also decreased (Figure 3B). As assessed by ELISA, TGF‐β1 exposure was also associated with decreased concentrations of BMP4 and IGFBP4 in medium conditioned by MCs (Figure 3C).

### Exogenous BMP4 or IGFBP4 ameliorates TGF‐β1‐induced MMT *in vitro*

**Figure 4:**
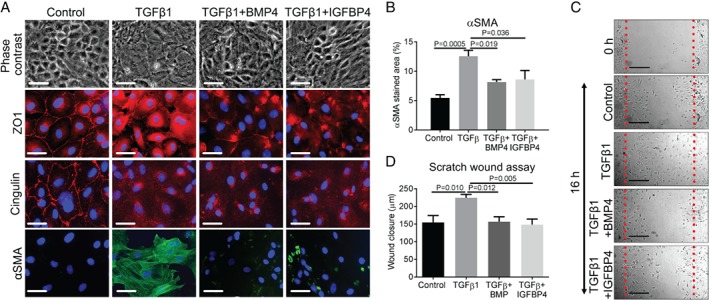
Application of BMP4 or IGFBP4 to TGF‐β1‐exposed rat MCs. (A) Cells were maintained for 48 h in either basal medium alone (control), medium supplemented with 1 ng/ml TGF‐β1, or the latter supplemented with either 50 ng/ml BMP4 (TGF‐β1 + BMP4) or 50 ng/ml IGFBP4 (TGF‐β1 + IGFBP4). Cells were imaged by phase contrast microscopy (top row) or, as shown in subsequent rows, by immunofluorescence for ZO1, cingulin, and αSMA, with all nuclei counterstained with 4′,6‐diamidino‐2‐phenylindole (blue). Note that exposure to either BMP4 or IGFBP4 partially preserved the cobblestone pattern of the monolayer, reduced the cytoplasmic localization of ZO1, and reduced the level of αSMA. In contrast, neither factor rescued the TGF‐β1‐induced disruption of cingulin. Scale bars: 50 μm. (B) Quantification of αSMA by immunofluorescence, showing increased immunostaining in TGF‐β1‐treated versus control cells (*n* = 5; mean ±SEM). There was a reduction in αSMA expression in cells treated with TGF‐β1 + BMP4 or TGF‐β1 + IGFBP4. (C) Phase contrast images showing MC migration into a wound over a period of 16 h under different conditions. Scale bars: 200 μm. (D) Quantification of MC migration under different conditions (*n* = 6; mean ±SEM). Note that TGF‐β1 exposure was associated with more extensive migration than in controls (*n* = 6), and that this effect was abrogated when either BMP4 or IGFBP4 was added with TGF‐β1.

We hypothesized that the diminished levels of BMP4 and IGFBP4 described above might modulate TGF‐β1‐induced MMT. We administered 50 ng/ml BMP4 or IGFBP4 recombinant proteins to MC cultures. As assessed by gross morphology and immunostaining for ZO1, cingulin, and αSMA, addition of either protein alone did not affect the phenotype of these cells (data not shown). In contrast, addition of either BMP4 or IGFBP4 to TGF‐β1‐exposed cells (Figure 4A) preserved aspects of the epithelial phenotype and ameliorated the mesenchymal phenotype, as assessed by: preservation of a cobblestone appearance; less prominent ZO1 cytoplasmic localization; and a reduction in αSMA immunostaining (Figure 4A), confirmed to be significant upon quantification (Figure 4B). In contrast, neither exogenous BMP4 nor IGFBP4 prevented the loss of cell–cell cingulin localization following addition of TGF‐β1 (Figure 4A). A key event during MMT is cell migration, so we studied this with a scratch assay in MC cultures (Figure 4C). MCs exposed to 1 ng/ml TGF‐β1 showed enhanced wound closure at 16 h, consistent with increased migration. Addition of IGFBP4 or BMP4 abrogated this effect (Figure 4D).

### Exploration of BMP4 in two mouse models of peritoneal fibrosis

**Figure 5:**
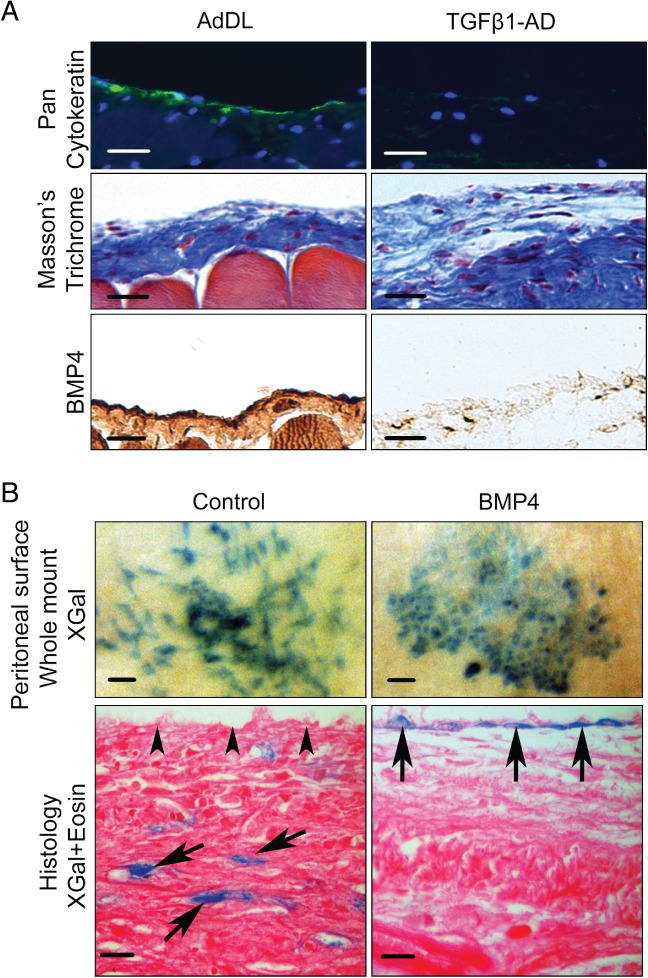
BMP4 in murine models of peritoneal fibrosis. (A) Immunostaining for cytokeratin in the peritoneum of mice showed diminished mesothelium‐specific expression in response to TGF‐β1 adenovirus (AD) as compared with control (AdDL); nuclei were stained with 4′,6‐diamidino‐2‐phenylindole. In response to TGF‐β1 overexpression, the peritoneum was extensively thickened, as shown by Masson's trichrome, with near‐complete loss of surface BMP4 immunostaining. Scale bars: 100 μm. (B) After surgical injury, the peritoneum of *Wt1* lineage tracing mice was stained with XGal. Cells expressing the *LacZ* reporter gene appear blue, and represent the fates of subsets of MCs and/or their progeny. The top two frames show whole mounts, looking down on the peritoneal surface. Injured mice that received vehicle alone (left frame) had elongated and spindle‐shaped labelled cells, whereas cobblestone‐like cell clusters were seen in similarly injured mice that had received BMP4 (right frame). Scale bars: 100 μm. The lower two frames show the histology of the peritoneum, with eosin (pink) counterstaining: the peritoneal surface is at the top. Injured mice receiving vehicle alone (left frame) showed labelled cells (arrows) below the surface (arrowheads) of the peritoneum. In injured mice that had received BMP4 (right frame), labelled cells (arrows) were noted on the surface of the peritoneum. Scale bars: 20 μm. Representative images from *n* = 3 in each group.

In a mouse model of peritoneal fibrosis induced by intraperitoneal TGF‐β1‐expressing adenovirus, there was attenuation of BMP4 immunostaining of the surface of the peritoneum (Figure 5A). Next, we ‘genetically labelled’ peritoneal MCs by activation of the *LacZ* allele induced by activating *Wt1* promoter‐driven Cre recombinase. Here, labelled cells and their progeny express a reporter that can be detected with the XGal reaction. As noted by Lua *et al*, Wt1 promoter‐driven Cre recombinase activation occurs in a subset of MCs, so only the fates of the labelled population can be tracked 18. Mice were subjected to surgery to induce adhesion formation, and a subset were treated with BMP4. In whole mount preparations, we found elongated blue cells in zones immediately adjacent to the nascent scar, whereas, in injured mice exposed to exogenous BMP4, clusters of cuboidal cells were noted (Figure 5B, upper frames). On histology, cells expressing the reporter were noted under the peritoneal surface after injury, whereas, after administration of BMP4, labelled cells were present on the peritoneal surface (Figure 5B, lower frames). These observations suggest that BMP4 helps to restore ‘healthy’ mesothelial morphology following injury *in vivo*.

### Altered patterns of BMP4 and IGFBP4 expression in human peritoneal dialysis tissue

**Figure 6:**
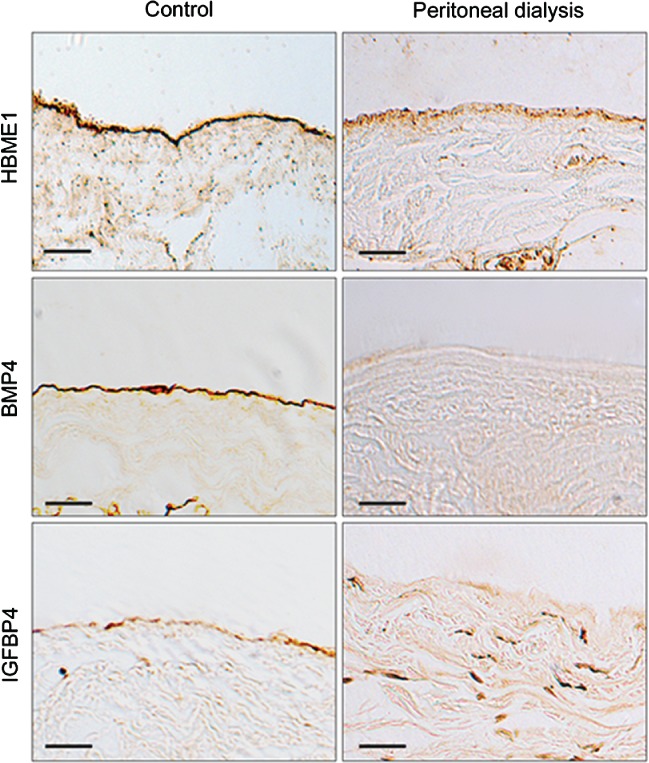
Human fibrotic tissue shows altered patterns of BMP4 and IGFBP4. Human control peritoneum or thickened peritoneum samples from peritoneal dialysis patients were immunostained for HBME1 to identify mesothelium. Control peritoneum showed prominent mesothelial IGFBP4 and BMP4 immunostaining, which was attenuated in peritoneal dialysis samples. In the peritoneal dialysis samples, scattered IGFBP4‐positive cells were noted below the peritoneal surface. Scale bar: 100 μm.

Mesothelium was identified in control and peritoneal dialysis‐exposed human peritoneal tissue sections, as determined by HBME1 immunostaining (Figure 6). Control peritoneal tissue, from otherwise healthy patients undergoing incidental hernia surgical repair, showed positive immunostaining for both IGFBP4 and BMP4 on the peritoneal surface. This pattern was attenuated in tissue harvested from peritoneal dialysis patients (Figure 6). Scattered cells below the mesothelial layer showed positive staining for IGFBP4, which may represent retention of some mesothelial characteristics in cells undergoing MMT 10.

## Discussion

Previous studies investigating peritoneal MCs generally analysed cultures obtained solely by enzymatic digestion of omentum 23, 33, 34. A few studies have enriched for MCs by using procedures such as positive selection for HBME1 with FACS 15, removal of CD45‐positive cells 35, or sorting for glycoprotein M6a (GpM6a)‐expressing cells with magnetic beads 18. We found similar levels of MC enrichment by FACS and magnetic bead sorting: both use positive sorting with HBME1 antibody against a microvillus protein characteristic of MCs and produced populations in which 90% of cells were Wt1‐positive. Magnetic bead sorting is a convenient method that bypasses the need for cell sorting facilities. Whether sorting by the use of both HBME1 and another specific MC marker, such as mesothelin or GpM6a, generates greater enrichment could be addressed in future studies.

Using RNA‐seq, we identified numerous ‘epithelial marker’ RNAs in purified rat MCs, including transcripts encoding ZO1, mesothelin, uroplakin 3B, and podoplanin, similarly to previous reports. Purified MCs also expressed high levels of transcripts for several keratins, *Wt1*, and *Msln*, encoding the cell surface protein mesothelin 26. MCs appear to share certain molecules, including Wt1 and podocalyxin‐like protein 1, with podocytes, which are specialized epithelial cells within kidney glomeruli. As for podocytes, the molecular signature of MCs has certain similarities to that of generic mesenchymal cells; for example, both epithelia contain abundant vimentin 36. Thus, MCs are ‘epithelial‐like’ rather than exactly like ‘classic’ epithelia.

The expression of the cell–cell junction protein E‐cadherin, and its downregulation, reflecting the destabilization of adherens junctions, has been considered to be a hallmark of EMT, and has been noted in some MMT studies 23, 35, 37. In our study, however, RNA‐seq of HBME1‐sorted rat MCs revealed very low read numbers for *Cdh1*, the transcript encoding E‐cadherin; moreover, there was no significant change after TGF‐β1 exposure. Furthermore, E‐cadherin was not detected in rat omentum by the use of immunohistochemistry. The literature already points to heterogeneity of E‐cadherin expression by MCs studied in different contexts. Cells harvested from human omentum, or collected from dialysis effluent, expressed E‐cadherin 35, but MCs covering the liver 38 or the body wall 18 of mice did not express E‐cadherin *in vivo*, and nor did human ovarian MCs 39.

Given the heterogeneity of MCs, depending on their source, there are unlikely to be exactly the same changes in gene expression as they undergo MMT. Ruiz‐Carpio *et al* 35 analysed human peritoneal cells that had undergone, or were undergoing, MMT. MMT was associated with increased levels of *THBS1*, *VCAN*, and *ITGA11*, whereas *BMP4* and *THBD* were downregulated. Inspection of our RNA‐seq data revealed similar significant changes. On the other hand, Ruiz‐Carpio *et al* noted markedly increased levels of *IL33* and *IL6*, whereas we found that the former transcript was expressed but unchanged after TGF‐β1, and the latter was not expressed. Moreover, Ruiz‐Carpio *et al* also noted markedly decreased levels of *AQP1*, *MUC16*, and *VTN*. In our arrays, the former two transcripts were expressed but unchanged by TGF‐β1, whereas the latter was not expressed. Moreover, because MCs are not typical epithelial cells, MMT molecular profiles are unlikely to be exactly the same as for EMT. In our RNA‐seq study, *Snai1*, *Snai2* and *Zeb2* were all significantly upregulated in TGF‐β1‐induced MMT, and the levels of all three tended to increase in typical EMT. Conversely, we did not detect TGF‐β1‐induced changes in either *Cdh2*, encoding the cell–cell junction protein N‐cadherin, or in *Twist1* and *Twist2*, encoding transcription factors: all three have been implicated in typical EMT. Collectively, these observations support the idea that that there are unlikely to be exactly the same changes in gene expression in all forms of EMT or MMT.

In the current study, *Sox9*, encoding sex‐determining region Y‐box 9, was significantly upregulated in MMT. This transcription factor has been implicated in fibrosis 40, and probably synergizes with SNAIL1 or SNAIL2 to drive EMT 41, 42. The role of Sox9 in regulating MMT and peritoneal fibrosis warrants further investigation. Wt1 is a transcription factor that regulates the balance between EMT and MET during development 43, 44. In the current study, purified MCs expressed high levels of *Wt1*, but these showed no significant change in response to TGF‐β1. Another group, analysing pleural MCs, detected MMT when Wt1 was experimentally downregulated 45. Thus, downregulation of Wt1 can be associated with MMT, but appears not to be essential for TGF‐β1‐induced MMT in our current study.

We also found that TGF‐β1 exposure led to upregulation of transcripts encoding tenascins C and N. Tenascin C is an extracellular matrix protein that has been found to inhibit cellular adhesion to fibronectin and has been recently proposed as a biomarker in peritoneal dialysis associated with poor membrane function 46. Tenascin N is a member of the tenascin family that has been previously associated with neurite outgrowth, and appears not to have been previously highlighted in MMT, so it is worthy of further investigation. Upregulation of neural cell adhesion molecule 1 has been reported to promote the formation of focal adhesions in mesothelium‐derived tumours 47, but a possible functional role in MMT is yet to be elucidated. Vascular cell adhesion molecule 1, which is important for leukocyte adhesion, was also induced by TGF‐β1; it has previously been reported to be upregulated in MCs exposed to advanced glycation end‐products 48, 49, and the soluble form has been reported to inhibit MMT 50.

We hypothesized that, if the molecular signature of MMT could be better defined, these insights could be exploited to block this pathological cellular transition. We discovered that, in response to TGF‐β1, MCs showed robust downregulation of BMP4. Apart from BMP1, which is a metalloprotease, BMPs are secreted growth factors belonging to the TGF‐β superfamily, which bind to dimers of BMP receptors I and II, eliciting intracellular signalling via SMAD phosphorylation. BMP4 is required for gastrulation and for lung, heart and kidney development 51. We reasoned that the TGF‐β1‐induced depletion of BMP4 might itself modulate MMT. Indeed, recombinant BMP4 partially prevented TGF‐β1‐induced MMT, as indicated by retention of membranous ZO1 localization, lack of αSMA induction and reduced cell migration *in vitro*. Moreover, we found that BMP4 immunostaining on peritoneal surfaces was attenuated versus healthy controls in human peritoneal dialysis tissue and in a mouse model of TGF‐β1‐induced peritoneal fibrosis. Finally, genetic lineage tracing of MCs was used in mice with peritoneal injury. Here, BMP4 administration ameliorated injury‐induced shape change and migration of cells expressing the reporter gene. We interpret these results as showing that genetically labelled MCs, and/or their progeny, move under the surface of the injured mesothelium, as also concluded by Lua *et al* when they examined a model of chlorhexidine gluconate‐induced fibrosis 18. Our findings demonstrate a distinctive MMT signature, and highlight the therapeutic potential for BMP4 to reduce MMT. Future experiments will be needed, however, to determine whether BMP4 ameliorates the degree of scarring *in vivo*. Although this is a novel finding with regard to MMT, application of BMP4 was shown to reduce retinal epithelial cell EMT in a similar manner 52. BMP7 has been shown to ameliorate MMT in previous studies, and BMP7 and BMP4 signal through similar pathways 53. Our RNA‐seq data showed that *Bmp4* was highly expressed in rat MCs, whereas *Bmp7* read counts were low; moreover, only *Bmp4* was significantly downregulated upon exposure to exogenous TGF‐β1 [*Bmp4* average reads of 3434 reducing to 1428 with exposure to TGF‐β1 (*p* = 5.09 × 10^–24^); and average *Bmp7* reads at baseline of 80, and 39 upon exposure to exogenous TGF‐β1 (*p* = 0.108). Therefore, of these two BMP molecules, it is BMP4 that is the key endogenous factor in our model. In parallel with the downregulation of BMP4 in MCs undergoing MMT, there was an increase in the level of the transcript encoding gremlin‐2, a member of the BMP‐antagonist gremlin family 29, 54. Notably, it has been reported that adenovirus‐mediated upregulation of *gremlin1* promotes peritoneal fibrosis in mice 55. We speculate that BMP4 signalling via phosphorylation of Smad1/5/8 opposes TGF‐β1‐mediated phosphorylation of Smad2/3 signalling 53. Thus, the two signalling pathways may maintain the balance of homeostasis in the mesothelium.

Our transcriptome analyses also revealed that transcripts encoding the growth factors IGF1 and IGF2 were upregulated during MMT. In parallel, the levels of transcripts encoding IGFBP4 and IGFBP5 were markedly downregulated. These changes are predicted to lead to an increase in IGF signalling activity, as IGFBPs lengthen the half‐life of circulating IGF1, owing to their higher affinity for IGF ligands than the receptors 31. We showed that recombinant IGFBP4, like BMP4, ameliorated TGF‐β1‐induced MMT *in vitro*. Furthermore, immunostaining for IGFBP4 of peritoneal tissues harvested from peritoneal dialysis patients was less prominent in the mesothelial layer, with some evidence of expression in cells of the submesothelium that might represent migrated MCs or their progeny. IGF signalling interacts at several levels with various components of the TGF‐β signalling pathway 56. In future, it will be important to determine whether similar interactions may be regulating IGF‐induced MMT in the peritoneum.

Blocking downstream TGF‐β1 signalling pathways may be another way to attenuate MMT; these include Smad‐dependent 57 and Smad‐independent pathways, such as Akt–mammalian target of rapamycin 57, c‐Jun N‐terminal kinase 57, Wnt–β‐catenin 58, integrin‐linked kinase–glycogen synthase kinase‐3β 59, extracellular signal‐regulated kinase–nuclear factor‐κB 33, and mitogen‐activated protein kinase 60. Another approach, however, based on the findings from the current study, would be to focus on introducing BMP4 and IGFBP4 to prevent MMT and peritoneal fibrosis. Importantly, evidence suggests MMT occurs in diverse peritoneal pathologies, including surgical adhesions 61, endometriosis 62, and peritoneal metastasis 63. In a rat model of surgical adhesions, IGFBP4 administration ameliorated peritoneal scarring 64, although a link to MMT was not explored. Future studies are required to elucidate whether therapeutically manipulating BMP4 or IGFBP4 signalling could ameliorate the severity of these conditions.

## Author contributions statement

SN contributed to conception and design, all aspects of data acquisition apart from the lineage tracing experiment, and the preparation of figures and drafting of the manuscript. SH and AW contributed to conception and design, data interpretation, and drafting of the manuscript. TW and BW designed and undertook the lineage tracing experiment, and revised the manuscript critically. LZ contributed to RNA‐seq data acquisition and preparation of figures. All authors were responsible for approval of the final manuscript.

**SUPPLEMENTARY MATERIAL ONLINE**

**Supplementary materials and methods**

**Supplementary figure legends**

**Figure S1.** E‐cadherin immunostaining of rat omental mesothelial cells

**Table S1.** Epithelial signature transcripts

**Table S2.** Mesenchymal/extracellular matrix transcripts

**Table S3.** Transcription and growth factors implicated in EMT and/or MMT

**Table S4.** Transcripts implicated in BMP and IGF signalling

## Supporting information


**Supplementary materials and methods**

**Supplementary figure legends**

**Figure S1. E‐cadherin immunostaining of rat omental mesothelial cells.** (A) Serial rat omental sections were immunostained for HBME1 and pan‐cytokeratin to identify the mesothelium and E‐cadherin. Note the absence of E‐cadherin immunostaining of the omental mesothelium. Sections of nearby rat pancreas used as a positive control were devoid of HBME1 but showed both cytokeratin and intense junctional E‐cadherin immunostaining. (B) Confluent monolayers of cultured sorted rat mesothelial cells showed little positive immunostaining for E‐cadherin. (Arrow indicates possible weak junctional staining.) In contrast, cultured human epithelial breast cancer cells, MCF7, displayed prominent junctional E‐cadherin staining. No primary acted as a negative control for tissue sections and cell cultures and nuclei were stained with DAPI. Scale bars are 100 μm. (C) Unsorted and Mojo‐sorted MCs displayed comparable CT values for E‐cadherin.

**Table S1.** Epithelial signature transcripts

**Table S2.** Mesenchymal/extracellular matrix transcripts

**Table S3.** Transcription and growth factors implicated in EMT and/or MMT

**Table S4.** Transcripts implicated in BMP and IGF signalling
