# Supplementary material for: Functional molecules in mesothelial‐to‐mesenchymal transition revealed by transcriptome analyses
Source: J Pathol. 2018 Jul 4;245(4):491–501. doi: 10.1002/path.5101 (PMC6055603; doi:10.1002/path.5101)
Supplement: Supplementary file 1 — Supplementary materials and methods [file PATH-245-491-s007.docx]

**Supplementary Material and Methods**

***RNA sequencing (RNA-seq) and quantitative polymerase chain reaction (QPCR)***

For RNA-seq, paired samples (n = 5) from control and TGFβ1-exposed MCs were collected in RNA protect (Thermo Fisher Scientific) and RNA extracted using the RNeasy Plus Mini Kit (Qiagen, Manchester UK). Libraries were generated with the TruSeq Stranded mRNA Library Prep Kit and sequenced paired-end on the Illumina HiSeq4000 platform with an average of 33 million reads per sample (ArrayExpress repository E-MTAB-5998). Sequences were tested by FastQC v0.11.5 using various metrics (http://www.bioinformatics.babraham.ac.uk/projects/fastqc/). Sequence adapters were removed and reads were quality trimmed using Trimmomatic v0.36 [65]. The reads were mapped against the reference rat genome, rn6 using STAR v2.4.2 [66]. Counts per gene were calculated with HTSeq v0.6.1 [67] using annotation from Ensembl v6.0.85. Normalisation and differential expression was calculated with DESeq2 v1.10.1, R v3.2.3 [68]. Differentially expressed transcripts were defined as those showing a log_2_(fold change) > 0.36 or log_2_(fold change) < −0.36 *vs.* controls, and a statistical significance of P < 0.05, corrected for multiple comparisons. For QPCR, cDNA was synthesised using the TaqMan Reverse Transcription Reagent kit (Thermo Fisher Scientific). QPCR was performed using the RotorGene 6000 (Qiagen) with 2 × SensiFAST SYBRGreen No-ROX (Bioline), cDNA template and rat specific primers (Primerdesign, Eastleigh, UK). Data was normalised to the housekeeping transcript *Gapdh* and analysed by the ΔΔC_T_ method. Note that, as assessed by RNA-seq, *Gapdh* levels showed no significant difference between control and TGFβ1-exposed MCs. The following forward and reverse primers were used:

| Gapdh | GACATGCCGCCTGGAGAAAC | AGCCCAGGATGCCCTTTAGT |
| --- | --- | --- |
| Vimentin | ATTTCTCTGCCTCTTCCAAACTT | CCGTCTTAATCAGGAGTGTTCTT |
| Podxl | CGCTTCAGCCTTCCTCTCAT | GCTCCTCTGTGAGTCGTTGT |
| Zo1 | AACCCGAAACTGATGCTATGG | CCTTGGAATGTATGTGGAGAGAA |
| Cgn | CTCTATCCAGATTGATGATGAACGG | CTTCTTCCTCAGGCTGTCCAG |
| Bmp4 | CCAAGCGTAGTCCCAAGCA | GCCACGATCCAATCATTCCAG |
| Igfbp4 | AACACCCTCCCTCTCAATGTG | GAGGACCTGAGGAATGACCTAC |
| Wt1 | GCCAGGATGTTCCCCAATG | CGAAAGTGACCGTGCTGTAT |
| Col4a3 | GCTGCCAGGACCAGTGATT | TGACCATAGGAGTCTCCAGGT |
| Col4a4 | AACTCGCAGCCAGCACAC | CAGAAGATTCTCATGGACAGTTGG |
| Up3b | ATGACACCATCTGGCTAGTGG | ATCTTAGCAGCGGTCTGTGG |
| Cdh1 | CATGAGTGTCCCCCGGTATC | CAGTATCAGCCGCTTTCAGA |

***Immunostaining rat tissues***

Cells were cultured on glass chamber slides (ThermoFisher). Paraformaldehyde (PFA)-fixed cultures were incubated for 30 min at room temperature with primary antibodies to: α-smooth muscle actin (α-SMA, 1:400; Cat No: A 2547, Sigma Aldrich); cingulin (1:100; Cat No: NBP1-89600, Bio-Techne, Abingdon, UK); E-cadherin (1:50; Cat No: ab76055, Abcam or 1:250; Cat No: BD610181, BD Biosciences) **;** HBME1 (1:50; Cat No: M3505; Dako); vimentin (1:100; Cat No: V 6630, Sigma Aldrich); Wilms tumour 1 (Wt1; 1:100; Cat No: SC-192, Santa Cruz, Heidelberg, Germany); or zonula occludens 1 (ZO1; 1:50; Cat No: 61-7300, Thermofisher). For E-cadherin, cultured human epithelial breast cancer cells (MCF7; ATCC, Teddington, UK) were used as a positive control. Primary antibodies were diluted in 3% BSA blocking buffer or 3% BSA supplemented with 0.1% Triton (Sigma-Aldrich), as indicated. Cells were exposed to secondary antibodies conjugated to Alexa488 or Alexa568 (1:400; Thermo Fisher Scientific) for 30 min and mounted in VectaShield media containing DAPI (Vector Laboratories, Cambridgeshire, UK). Fluorescence was visualised using a BX51 upright microscope (Olympus) and captured using a Coolsnap ES2 camera (Photometrics). To quantify fluorescence, at least four fields of view per well were analysed and percentage area of staining was calculated with Fiji software. Because of the punctate appearance of HBME1 immunostaining in cultured MCs, the number of positive pixels (raw integrated density) of HBME1 immunostaining was factored for the number of DAPI-positive nuclei in each field of view. For analysis of Wt1 immunostaining in cells, the percentage of positive nuclei was determined in Image J. For rat omentum and pancreas immunohistochemistry, 15-μm frozen sections were fixed in 4% PFA and immunostained with primary antibodies against HBME1, cytokeratin (Cat No: C1801, Sigma) or E-cadherin for 48 h at 4˚C and processed for immunostaining as for cultured cells. Tissue sections were imaged by confocal microscopy (Leica TCS SP5 AOBS).

***Cell migration assay***

A cell culture scratch assay was used where MC monolayers were cultured in media containing 5% FCS overnight, before a scratch was created across the centre of the wellusing a 200-μl pipette tip. Live cell imaging was performed in a humidified chamber at 37°C and 5% CO_2_. Images were obtained every 20 min over 18 h for at least three fields of view per well, with at least three wells per condition, using an AS MDW live cell imaging system (Leica) and imaging software Micromanager MM Studio 1.4.20 at × 10 magnification.

***ELISA***

Concentrations of rat BMP4 (CUSABIO ELISA kit, Hubei province, China) and rat IGFBP4 (US Biological ELISA kit, Salem, USA) in culture supernatants were assessed by ELISA according to manufacturers’ protocol. Briefly, 100 μl of supernatant was tested in duplicate and optical density values were used to interpolate values from each standard curve. The lower limit of detection for the BMP4 ELISA was 1.95 pg/ml and for the IGFBP4 ELISA was 156 pg/ml.

***TGFβ1-induced peritoneal fibrosis and peritoneal MC lineage tracing in mice***

Male C57/BL6J mice aged 8 weeks (Charles River, Harlow, UK) were maintained in SPF conditions with food and water available *ad libitum*. Following acclimatisation, mice received a single intraperitoneal injection of 1.5 × 10^8^ pfu of a first-generation adenovirus expressing the active form of TGFβ1 (AdTGFβ1; kind gift of P. Margetts, McMaster University, Hamilton, Canada) in 100 μl of PBS (n = 5) as previously described [[16](file:///F:\MRC\J%20Pathology%20rebuttal\Figures%20150218\Namvar%20J%20Path%20revision%2027-2-18.docx#_ENREF_16)]. Control mice received 1.5 × 10^8^ pfu of a control adenovirus (AdDL) that lacked transgene expression (n = 5). After 7 days, the entire anterior abdominal wall was resected and the upper portion of tissue was PFA-fixed, processed and 7-µm sections collected. Sections were stained with Masson's trichrome or immunostained with primary antibody for pan-cytokeratin (C1801, Sigma). Next, we combined physical injury to the peritoneum by surgical abrasion of adjacent serosa followed by close apposition, as we previously described [[17](file:///F:\MRC\J%20Pathology%20rebuttal\Figures%20150218\Namvar%20J%20Path%20revision%2027-2-18.docx#_ENREF_17)] with a mesothelial lineage tracing strategy, similar to that described by Lua et al. but using *LacZ* rather than *GFP*, with induced peritoneal injury [[18](file:///F:\MRC\J%20Pathology%20rebuttal\Figures%20150218\Namvar%20J%20Path%20revision%2027-2-18.docx#_ENREF_18)]. Compound mutant mice *Wt1^tm2(cre/ERT2)Wtp/+^;B6.129S4-Gt(ROSA)26Sor^tm1Sor/^* [69, 70], aged 6-8 weeks were administered tamoxifen (Sigma) dissolved in corn oil (10 mg/ml; Sigma) at 1 mg/10 g body weight by oral gavage on 5 consecutive days, followed by 2 weeks washout according to published protocols [70]. Peritoneal adhesion formation was induced by local physical injury under surgery as we previously described [17]. At surgery and at day 1, 3 and 5 thereafter, mouse BMP4 (recombinant carrier-free, BioLegend) reconstituted in 10 mM citric acid was injected intraperitoneally at 300 ng/g body weight using medical-grade saline (50 ng/µl, 0.1% BSA; n = 3), while control animals received vehicle saline alone (containing 0.1% BSA and citric acid; n = 3). One week after BMP4 or vehicle control administration, caecum-peritoneal wall adhesions and surrounding tissue was collected. Tissue was fixed in 2% PFA, 0.2% glutaraldehyde, followed by whole mount XGal staining according to published protocols [70]. Images were captured using a Leica DFC420C camera attached to a Leica MZ16F dissection microscope. Regions containing XGal-positive cells in the adhesion zone were dissected and processed into Eosin-stained paraffin sections. Images were captured using a Leitz DM RB microscope with a Leica DFC 450C camera.
