# Supplementary material for: Functional molecules in mesothelial‐to‐mesenchymal transition revealed by transcriptome analyses
Source: J Pathol. 2018 Jul 4;245(4):491–501. doi: 10.1002/path.5101 (PMC6055603; doi:10.1002/path.5101)
Supplement: Supplementary file 2 — Supplementary figure legends [file PATH-245-491-s004.docx]

**Figure S1. E-cadherin immunostaining of rat omental mesothelial cells.** (A) Serial rat omental sections were immunostained for HBME1 and pan-cytokeratin to identify the mesothelium and E-cadherin. Note the absence of E-cadherin immunostaining of the omental mesothelium. Sections of nearby rat pancreas used as a positive control were devoid of HBME1 but showed both cytokeratin and intense junctional E-cadherin immunostaining. (B) Confluent monolayers of cultured sorted rat mesothelial cells showed little positive immunostaining for E-cadherin. (Arrow indicates possible weak junctional staining.) In contrast, cultured human epithelial breast cancer cells, MCF7, displayed prominent junctional E-cadherin staining. No primary acted as a negative control for tissue sections and cell cultures and nuclei were stained with DAPI. Scale bars are 100 μm. (C) Unsorted and Mojo-sorted MCs displayed comparable CT values for E-cadherin.
