# Supplementary figures and images for: Functional molecules in mesothelial‐to‐mesenchymal transition revealed by transcriptome analyses
Source: J Pathol. 2018 Jul 4;245(4):491–501. doi: 10.1002/path.5101 (PMC6055603; doi:10.1002/path.5101)

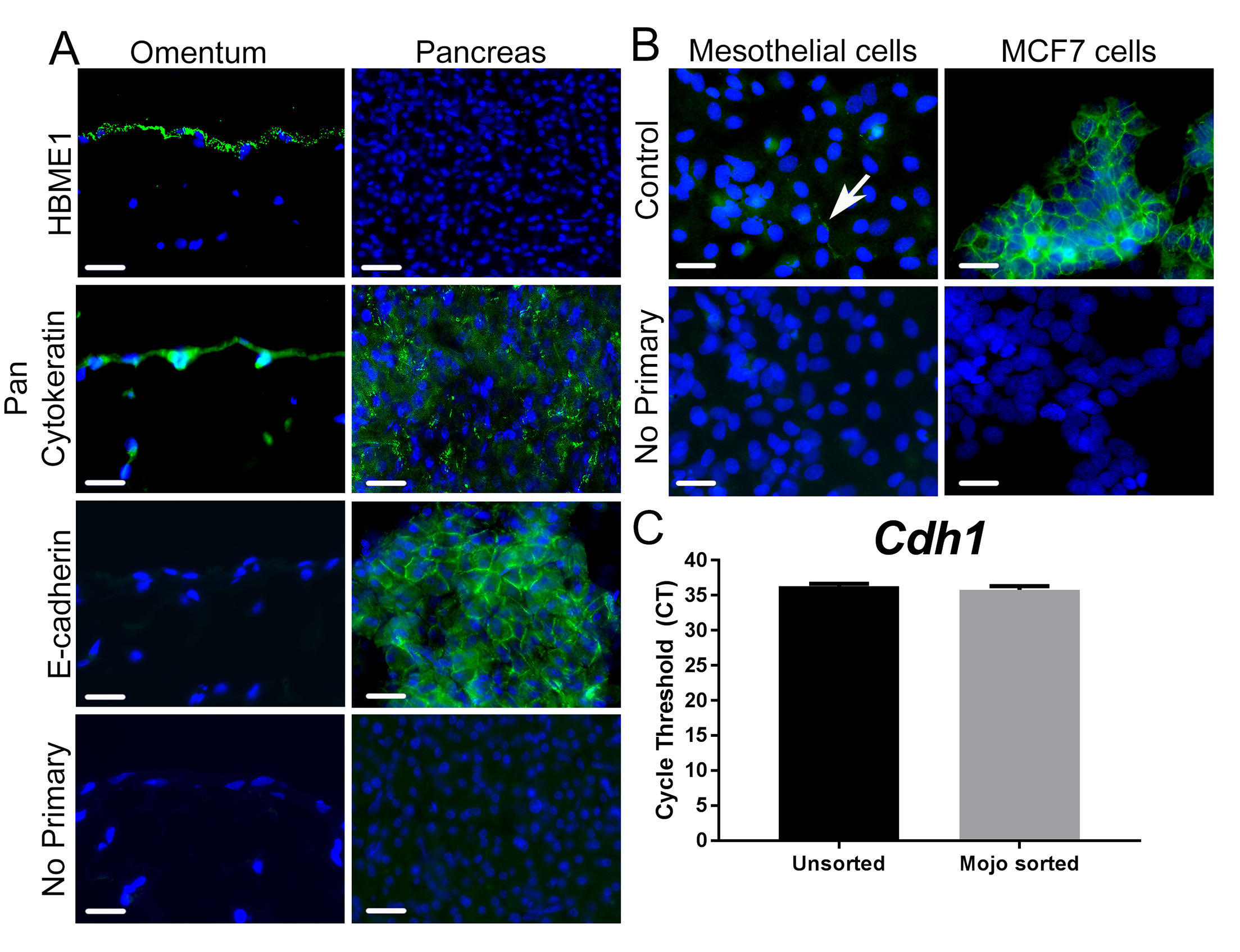

Supplement: Supplementary file 3 — Figure S1. E‐cadherin immunostaining of rat omental mesothelial cells. (A) Serial rat omental sections were immunostained for HBME1 and pan‐cytokeratin to identify the mesothelium and E‐cadherin. Note the absence of E‐cadherin immunostaining of the omental mesothelium. Sections of nearby rat pancreas used as a positive control were devoid of HBME1 but showed both cytokeratin and intense junctional E‐cadherin immunostaining. (B) Confluent monolayers of cultured sorted rat mesothelial cells showed little positive immunostaining for E‐cadherin. (Arrow indicates possible weak junctional staining.) In contrast, cultured human epithelial breast cancer cells, MCF7, displayed prominent junctional E‐cadherin staining. No primary acted as a negative control for tissue sections and cell cultures and nuclei were stained with DAPI. Scale bars are 100 μm. (C) Unsorted and Mojo‐sorted MCs displayed comparable CT values for E‐cadherin. [file PATH-245-491-s001.tif]
