# Supplementary material for: Functional molecules in mesothelial‐to‐mesenchymal transition revealed by transcriptome analyses
Source: J Pathol. 2018 Jul 4;245(4):491–501. doi: 10.1002/path.5101 (PMC6055603; doi:10.1002/path.5101)
Supplement: Supplementary file 4 — Table S1. Epithelial signature transcripts [file PATH-245-491-s005.docx]

**Table S1. Epithelial signature transcripts**

| **Gene Symbol** | **Encoded molecule** | **Control mean reads** | **TGFβ1 mean reads** | **log_2_(fold change) (paired)** | **FDR** |
| --- | --- | --- | --- | --- | --- |
| ***Down-regulated genes*** | | | | | |
| *Cgn* | Cingulin | 1559 | 560 | -1.507 | 2.03E-26 |
| *Podxl* | Podocalyxin-like | 6607 | 3107 | -1.163 | 1.09E-11 |
| *Cldn15* | Claudin 15 | 9166 | 5886 | -0.654 | 1.14E-09 |
| *Cldn2* | Claudin 2 | 1427 | 781 | -0.797 | 4.41E-08 |
| *Col4a3* | Collagen 2C type IV | 488 | 248 | -1.186 | 2.38E-07 |
| *Upk3b* | Uroplakin 3B | 20429 | 14864 | -0.467 | 3.73E-06 |
| *Col4a4* | Collagen 2C_type_IV 2C_alpha_4 | 11887 | 5809 | -1.265 | 4.03E-06 |
| *Krt23* | Keratin_23 | 135 | 48 | -1.388 | 5.08E-06 |
| *Krt13* | Keratin_13 | 426 | 217 | -1.250 | 6.08E-06 |
| *Tjp1* | Tight_junction_protein_1 (ZO-1) | 15384 | 11569 | -0.416 | 3.38E-05 |
| *Itgb3* | Integrin_subunit_beta_3 | 689 | 396 | 0.682 | 9.00E-05 |
| *Ppl* | Periplakin | 5588 | 2588 | -1.160 | 0.000133 |
| *Itga6* | Integrin_subunit_alpha_6 | 1325 | 774 | -0.842 | 0.000297 |
| *Lamb2* | Laminin_subunit_beta_2 | 6366 | 4755 | -0.423 | 0.001374 |
| *Itgb4* | Integrin_subunit_beta_4 | 1367 | 975 | -0.500 | 0.001535 |
| *Krt19* | Keratin_19 | 463 | 216 | -1.581 | 0.00259 |
| *Krt18* | Keratin_18 | 350 | 232 | -0.727 | 0.002744 |
| *Lamb3* | Llaminin_subunit_beta_3 | 35 | 13 | -1.399 | 0.007653 |
| *Cldn1* | Claudin_1 | 5787 | 4347 | -0.445 | 0.015106 |
| *Krtap17-1* | Keratin_associated_protein_17-1 | 12 | 2 | -2.480 | 0.033379 |
| *Lyve1* | Lymphatic_vessel_endothelial_hyaluronan_receptor_1 | 1279 | 233 | -1.514 | 0.036201 |
| ***Unaltered genes*** | | | | | |
| *Cdh1* | Cadherin_1 | 20 | 26 | 0.673 | 0.441269 |
| *Wt1* | Wilms_tumor_1 | 10613 | 10041 | -0.091 | 0.588825 |
| *Msln* | Mesothelin | 63897 | 62175 | -0.046 | 0.8759 |
| ***Up-regulated genes*** | | | | | |
| *Sfn* | Stratifin | 66 | 472 | 2.802 | 1.53E-33 |
| *Lamc2* | Laminin_subunit_gamma_2 | 180 | 245 | 0.450 | 0.014634 |

Selected ‘epithelial’ transcripts, with mean number of reads in control and TGFβ1-exposed MCs, along with log_2_(fold change) and P values corrected for false discovery rate (FDR).
