# Supplementary material for: Functional molecules in mesothelial‐to‐mesenchymal transition revealed by transcriptome analyses
Source: J Pathol. 2018 Jul 4;245(4):491–501. doi: 10.1002/path.5101 (PMC6055603; doi:10.1002/path.5101)
Supplement: Supplementary file 5 — Table S2. Mesenchymal/extracellular matrix transcripts [file PATH-245-491-s006.docx]

**Table S2. Mesenchymal/extracellular matrix transcripts**

| **Gene Symbol** | **Encoded molecule** | **Control mean reads** | **TGFβ1 mean reads** | **log_2_(fold change) (paired)** | **FDR** |
| --- | --- | --- | --- | --- | --- |
| ***Up-regulated genes*** | | | | | |
| *Ncam1* | Neural_cell_adhesion_molecule_1 | 3953 | 12859 | 1.697 | 5.70E-86 |
| *Vcam1* | Vascular_cell_adhesion_molecule_1 | 228 | 1520 | 2.655 | 1.76E-54 |
| *Tnc* | Tenascin_C | 2051 | 23742 | 3.411 | 1.10E-24 |
| *Myh10* | Myosin 2C_heavy_chain_10 2C_non-muscle | 11788 | 18601 | 0.657 | 8.78E-24 |
| *Tnn* | Tenascin_N | 36 | 1107 | 4.501 | 8.21E-21 |
| *Col4a1* | Collagen 2C_type_IV 2C_alpha_1 | 77636 | 135323 | 0.846 | 5.64E-19 |
| *Itga8* | Integrin_subunit_alpha_8 | 3040 | 7398 | 1.261 | 4.60E-18 |
| *Vcl* | Vinculin | 5691 | 9826 | 0.776 | 8.38E-16 |
| *Fscn1* | Fascin_actin-bundling_protein_1 | 1041 | 2146 | 1.043 | 5.51E-13 |
| *Col4a2* | Collagen 2C_type_IV 2C_alpha_2 | 46061 | 67902 | 0.587 | 2.61E-12 |
| *Col5a1* | Collagen 2C_type_V 2C_alpha_1 | 32014 | 53822 | 0.714 | 2.27E-11 |
| *Nexn* | Nexilin_(F_actin_binding_protein) | 168 | 474 | 1.521 | 2.76E-11 |
| *Msn* | Moesin | 15597 | 24472 | 0.649 | 2.60E-10 |
| *Col1a1* | Collagen 2C_type_I 2C_alpha_1 | 394092 | 603398 | 0.614 | 6.44E-10 |
| *Tns1* | Tensin_1 | 10590 | 14483 | 0.456 | 9.23E-10 |
| *Vim* | Vimentin | 44279 | 68467 | 0.607 | 5.02E-09 |
| *Myh11* | Myosin 2C_heavy_chain_11 2C_smooth_muscle | 302 | 797 | 1.225 | 8.56E-08 |
| *Tagln* | Transgelin | 12509 | 47656 | 1.871 | 1.13E-07 |
| *Itga11* | Iintegrin_subunit_alpha_11 | 457 | 1006 | 0.987 | 6.25E-07 |
| *Col5a2* | Collagen 2C_type_V%2C_alpha_2 | 82389 | 108911 | 0.401 | 3.98E-06 |
| *Itgb1* | Integrin_subunit_beta_1 | 78702 | 105859 | 0.415 | 1.32E-05 |
| *Acta1* | Actin 2C_alpha_1 2C_skeletal_muscle | 5 | 41 | 2.685 | 3.50E-05 |
| *Itga5* | Integrin_subunit_alpha_5 | 4444 | 6242 | 0.497 | 0.000182 |
| *Vcan* | Versican | 4112 | 7823 | 1.073 | 0.000438 |
| *Itgae* | Integrin_subunit_alpha_E | 147 | 228 | 0.629 | 0.00048 |
| *Itgb6* | Integrin_subunit_beta_6 | 10 | 32 | 1.673 | 0.001715 |
| *Col3a1* | Collagen 2C_type_III 2C_alpha_1 | 378113 | 468616 | 0.303 | 0.001948 |
| *Des* | Desmin | 5410 | 6856 | 0.347 | 0.002501 |
| *Acta2* | Actin 2C_alpha_2 2C_smooth_muscle 2C_aorta (α-sma) | 5648 | 22466 | 1.970 | 0.003539 |
| *Cib2* | Calcium_and_integrin_binding_family_member_2 | 235 | 337 | 0.514 | 0.006403 |
| *Itgav* | Integrin_subunit_alpha_V | 3648 | 4592 | 0.351 | 0.013587 |
| *Itga10* | Integrin_subunit_alpha_10 | 3 | 14 | 2.126 | 0.01948 |
| *Itgbl1* | Integrin_subunit_beta_like_1 | 5767 | 7379 | 0.389 | 0.021021 |
| *Itga1* | Integrin_subunit_alpha_1 | 794 | 1035 | 0.382 | 0.03803 |
| ***Unaltered genes*** | | | | | |
| *F13a1* | Coagulation_factor_XIII_A1_chain | 3158 | 818 | -1.105 | 0.125258 |
| *Cdh2* | Cadherin_2 | 4330 | 4467.3 | 0.014 | 0.979014 |
| ***Down-regulated genes*** | | | | | |
| *S100a4* | S100_calcium-binding_protein_A4 (FSP1) | 10232 | 6267 | -0.730 | 1.33E-11 |

Selected ‘mesenchymal’ and extracellular matrix molecule transcripts, with mean number of reads in control and TGFβ1-exposed MCs, along with log_2_(fold change) and P values corrected for false discovery rate (FDR).
