# Supplementary material for: Functional molecules in mesothelial‐to‐mesenchymal transition revealed by transcriptome analyses
Source: J Pathol. 2018 Jul 4;245(4):491–501. doi: 10.1002/path.5101 (PMC6055603; doi:10.1002/path.5101)
Supplement: Supplementary file 6 — Table S3. Transcription and growth factors implicated in EMT and/or MMT [file PATH-245-491-s002.docx]

**Table S3. Transcription and growth factors previously implicated in EMT and/or MMT**

| **Gene Symbol** | | **Encoded molecule** | **Control mean reads** | **TGFβ1 mean reads** | **log_2_(fold change) (paired)** | **FDR** |
| --- | --- | --- | --- | --- | --- | --- |
| ***Up-regulated genes*** | | | | | | |
| *Postn* | Periostin | | 907 | 12663 | 3.933 | 4.53E-37 |
| *Tgfb2* | Transforming_growth_factor 2C_beta_2 | | 1168 | 2590 | 1.123 | 1.17E-23 |
| *Wisp1* | WNT1_inducible_signaling_pathway_protein_1 | | 2311 | 3982 | 0.803 | 8.75E-17 |
| *Mmp2* | Matrix_metallopeptidase_2 | | 8079 | 13357 | 0.694 | 9.05E-16 |
| *Timp1* | TIMP_metallopeptidase_inhibitor_1 | | 6227 | 10470 | 0.710 | 7.29E-13 |
| *Wnt5a* | Wingless-type_MMTV_integration_site_family 2C_member_5A | | 1322 | 2643 | 0.966 | 9.07E-13 |
| *Snai1* | Snail_family_transcriptional_repressor_1 | | 733 | 1275 | 0.757 | 2.66E-11 |
| *Tgfb1i1* | Transforming_growth_factor_beta_1_induced_transcript_1 | | 1173 | 2006 | 0.794 | 1.61E-08 |
| *Wnt2* | Wingless-type_MMTV_integration_site_family_member_2 | | 261 | 442 | 0.737 | 8.48E-08 |
| *Tgfb1* | Transforming_growth_factor 2C_beta_1 | | 798 | 1285 | 0.677 | 4.76E-07 |
| *Fndc3b* | Fibronectin_type_III_domain_containing_3B | | 4264 | 5293 | 0.311 | 7.17E-06 |
| *Sparc* | Secreted_protein_acidic_and_cysteine_rich | | 266980 | 341541 | 0.355 | 3.83E-05 |
| *Sox9* | SRY_box_9 | | 14 | 92 | 2.186 | 4.35E-05 |
| *Tgfb3* | Transforming_growth_factor 2C_beta_3 | | 8046 | 11071 | 0.440 | 7.64E-05 |
| *Snai2* | Snail_family_transcriptional_repressor_2 | | 125 | 232 | 0.866 | 7.85E-05 |
| *Wnt11* | Wingless-type_MMTV_integration_site_family 2C_member_11 | | 290 | 462 | 0.771 | 0.00019 |
| *Thbs1* | Thrombospondin_1 | | 8482 | 27041 | 1.534 | 0.000224 |
| *Mmp19* | Matrix_metallopeptidase_19 | | 1735 | 2187 | 0.327 | 0.000827 |
| *Zeb2* | Zinc_finger_E-box_binding_homeobox_2 | | 1129 | 1412 | 0.314 | 0.000898 |
| *Cemip* | Cell_migration-inducing_hyaluronan_binding_protein | | 11610 | 28004 | 1.630 | 0.004018 |
| *Plat* | Plasminogen_activator 2C_tissue_type | | 9468 | 11604 | 0.304 | 0.015156 |
| *Serpine1* | Serpin_family_E_member_1 | | 25951 | 84335 | 1.824 | 0.032918 |
| ***Unaltered genes*** | | | | | | |
| *Twist1* | Twist_family_bHLH_transcription_factor_1 | | 246 | 349 | 0.488 | 0.067817 |
| *Ctnnb1* | Catenin_beta_1 | | 7810 | 8552 | 0.131 | 0.139767 |
| *Twist2* | Twist_family_bHLH_transcription_factor_2 | | 65 | 95 | 0.534 | 0.17583 |
| *Ctnna1* | Catenin_alpha_1 | | 7268 | 7430 | 0.033 | 0.979014 |
| ***Down-regulated genes*** | | | | | | |
| *Thbd* | Thrombomodulin | | 3755 | 1352 | -1.451 | 1.37E-33 |
| *Timp4* | Tissue_inhibitor_of_metalloproteinase_4 | | 263 | 118 | -1.109 | 3.41E-08 |
| *Wnt2b* | Wingless-type_MMTV_integration_site_family 2C_member_2B | | 6478 | 4378 | -0.606 | 5.05E-08 |
| *Fgf1* | Fibroblast_growth_factor_1 | | 11522 | 6146 | -1.101 | 0.000169 |
| *Timp2* | TIMP_metallopeptidase_inhibitor_2 | | 40410 | 30888 | -0.375 | 0.000923 |
| *Zeb1* | Zinc_finger_E-box_binding_homeobox_1 | | 836 | 686 | -0.288 | 0.035739 |

Selected transcripts for growth factors and transcription factors previously implicated in EMT and/or MMT. Table contains mean number of reads in control and TGFβ1 exposed MCs along with log_2_(fold change) and P values corrected for false discovery rate (FDR).
