# Supplementary material for: Functional molecules in mesothelial‐to‐mesenchymal transition revealed by transcriptome analyses
Source: J Pathol. 2018 Jul 4;245(4):491–501. doi: 10.1002/path.5101 (PMC6055603; doi:10.1002/path.5101)
Supplement: Supplementary file 7 — Table S4. Transcripts implicated in BMP and IGF signalling [file PATH-245-491-s003.docx]

**Table S4. Transcripts implicated in BMP and IGF signalling**

| **Gene Symbol** | **Encoded molecule** | **Control mean reads** | **TGFβ1 mean reads** | **log_2_(fold change) (paired)** | **FDR** |
| --- | --- | --- | --- | --- | --- |
| ***Up-regulated genes*** | | | | | |
| *Igf1* | Insulin-like_growth_factor_1 | 4217 | 8274 | 0.953 | 1.52E-29 |
| *Pappa* | Pregnancy-associated_plasma_protein_A | 127 | 414 | 1.642 | 2.06E-22 |
| *Bmp1* | Bone_morphogenetic_protein_1 | 4437 | 6267 | 0.499 | 1.51E-11 |
| *Grem2* | Gremlin_2 2C_DAN_family_BMP_antagonist | 535 | 1340 | 1.297 | 1.07E-11 |
| *Igfbp7* | Insulin-like_growth_factor_binding_protein_7 | 28855 | 34731 | 0.269 | 0.000225 |
| *Igf2* | Insulin-like_growth_factor_2 | 62 | 118 | 1.437 | 0.000761 |
| ***Unaltered genes*** | | | | | |
| *Bmp7* | Bone_morphogenetic_protein_7 | 80 | 39 | -0.929 | 0.108664 |
| ***Down-regulated genes*** | | | | | |
| *Igfbp4* | Insulin-like_growth_factor_binding_protein_4 | 14736 | 3884 | -1.951 | 3.62E-50 |
| *Bmp4* | Bone_morphogenetic_protein_4 | 3434 | 1428 | -1.370 | 5.09E-24 |
| *Igfbp6* | Insulin-like_growth_factor_binding_protein_6 | 9830 | 6109 | -0.673 | 2.20E-12 |
| *Igfbp5* | Insulin-like_growth_factor_binding_protein_5 | 22768 | 9320 | -1.454 | 4.32E-07 |
| *Igfbp2* | Insulin-like_growth_factor_binding_protein_2 | 41556 | 29962 | -0.502 | 2.27E-05 |

Selected novel transcripts from BMP and IGF signalling pathways implicated in MMT. Table contains mean number of reads in control and TGFβ1 exposed MCs, along with log_2_(fold change).
